# A systematic review: efficacy of botulinum toxin in walking and quality of life in post-stroke lower limb spasticity

**DOI:** 10.1186/s13643-017-0670-9

**Published:** 2018-01-05

**Authors:** Anupam Datta Gupta, Wing Hong Chu, Stuart Howell, Subhojit Chakraborty, Simon Koblar, Renuka Visvanathan, Ian Cameron, David Wilson

**Affiliations:** 1 Department of Rehabilitation Medicine, The Queen Elizabeth Hospital, 28 Woodville Road, Adelaide, South Australia 5011 Australia; 2 School of Medicine, University of Adelaide, Adelaide, South Australia Australia; 3 Data, Design and Statistics Service, University of Adelaide, Adelaide, South Australia 5005 Australia; 4 John van Geest Centre for Brain Repair, Robinson Way, Cambridge, CB2 OPY UK; 5 South Australian Health and Medical Research Institute (SAHMRI), GPO Box 11060, Adelaide, South Australia 5001 Australia; 6 The Queen Elizabeth Hospital, 28 Woodville Road, Adelaide, South Australia 5011 Australia; 7 Head John Walsh Centre for Rehabilitation Research, Sydney Medical School, University of Sydney, Sydney, NSW 2006 Australia; 8 Department of Medicine, University of Adelaide, Adelaide, South Australia 5011 Australia

**Keywords:** Stroke, Spasticity, Botulinum toxin, Lower limb

## Abstract

**Background:**

Improved walking is one of the highest priorities in people living with stroke. Post-stroke lower limb spasticity (PSLLS) impedes walking and quality of life (QOL). The understanding of the evidence of improved walking and QOL following botulinum toxin (BoNTA) injection is not clear. We performed a systematic review of the randomized control trials (RCT) to evaluate the effectiveness of BoNTA injection on walking and QOL in PSLLS.

**Methods:**

We searched PubMed, Web of Science, Embase, CINAHL, ProQuest Thesis and Dissertation checks, Google Scholar, WHO International Clinical Trial Registry Platform, ClinicalTrials.gov, Cochrane, and ANZ and EU Clinical Trials Register for RCTs looking at improvement in walking and QOL following injection of BoNTA in PSLLS. The original search was carried out prior to 16 September 2015. We conducted an additional verifying search on CINHAL, EMBASE, and MEDLINE (via PubMed) from 16 September 2015 to 6 June 2017 using the same clauses as the previous search. Methodological quality of the individual studies was critically appraised using Joanna Briggs Institute’s instrument. Only placebo-controlled RCTs looking at improvement in walking and QOL were included in the review.

**Results:**

Of 2026 records, we found 107 full-text records. Amongst them, we found five RCTs qualifying our criteria. No new trials were found from the verifying search. Two independent reviewers assessed methodological validity prior to inclusion in the review using Joanna Briggs Institute’s appraisal instrument. Two studies reported significant improvement in gait velocity (*p* = 0.020) and < 0.05, respectively. One study showed significant improvement in 2-min-walking distance (*p* < 0.05). QOL was recorded in one study without any significant improvement. Meta-analysis of reviewed studies could not be performed because of different methods of assessing walking ability, small sample size with large confidence interval and issues such as lack of power calculations in some studies. Findings from our systematic and detailed study identify the need for a well-designed RCT to adequately investigate the issues highlighted.

**Conclusions:**

This review could not conclude there was sufficient evidence to support or refute improvement on walking or QOL following BoNTA injection. Reasons for this are discussed, and methods for future RCTs are developed.

## Background

Stroke is a common cause of adult disability worldwide [1]. More than two thirds of the stroke survivors develop post-stroke sequelae including impaired motor functions and spasticity [2]. The prevalence of post-stroke spasticity ranges from 19.0 to 42.6% [3]. There have been many recent developments in diagnosis, management, and prevention of stroke, while advances in rehabilitation have been modest [4]. There has, however, been progress with the use of botulinum toxin (BoNTA) as a treatment to improve spasticity in the upper limb [5–7]. Three systematic reviews [8–10] have addressed research progress on both the upper and lower limbs, with the conclusion from two of these that the effect on the upper and lower limbs spasticity favored BoNTA [8, 9]; however, these reviews did not fulfill the criteria for inclusion in this study.

As far as the lower limb is concerned, improvement in spasticity while important is only a first stage in post-stroke improvement, and the aim of RCTs should be to address the more important questions of functional activity including walking. How well this outcome has been addressed is the aim of this study. This is also an important question for many countries to resolve, because to date, botulinum toxin A is not approved for use in the post-stroke lower limb spasticity (PSLLS) by the pharmaceutical authorities except in the USA [11].

Lower limb spasticity most commonly involves the foot and the ankle leading to equinovarus (plantarflexion and inversion) deformity. Post-stroke patients with equinovarus deformity fail to achieve optimal contact with the ground leading to a poor stance, loss of heel to toe rhythm while walking and post-stroke patients walk predominantly with plantarflexion/inversion of the foot. Transfers and walking are essentially bipedal activity involving phases like balancing on one leg and swinging the other leg forward. The awkward position of the foot in addition to spasticity impairs balance, transfer, stride, gait, and mobility, besides causing spasm and pain. In many cases, complications like falls, fractures, deep vein thrombosis, and pressure ulcers may also result [12]. Inability to walk is associated with loss of independence and premature residential aged care placement [13, 14] and in the older population contributes substantially to adverse health outcomes including activities of daily living and mortality [15]. Improving and maintaining walking ability and activities of daily living are therefore vital for post-stroke survivors [16] and a major contributor to functional improvements. The overall human and economic cost of spasticity is, therefore, considerable, and interventions potentially can deliver significant benefits [17].

Given the evidence for efficacy of BoNTA in reducing spasticity, the objective of this review was to assess the available evidence of BoNTA injection: (1) to improve mobility (using gait velocity and walking distance as measuring parameters) and quality of life (QOL) and (2) to make appropriate recommendations for further research regarding these questions.

## Methods

### Review searches

The protocol used for this systematic review has been previously published [18]. In review searches, this review considered components of the protocol included in the literature search strategy of the studies, screening criteria and data extraction methods, assessment of methodological quality, and data collection and synthesis of data. Briefly, the literature search was performed on PubMed, Web of Science, EMBASE, CINAHL, ProQuest Thesis and Dissertation checks, and Google Scholar to identify RCTs prior to 16 September 2015. Medical subject headings and their indexing counterparts including “botulinum toxin,” “stroke,” and “muscle spasticity” were combined to search these databases, with filter settings for humans and English language activated. Detailed description of the search strategies for PubMed and Web of Science is provided in Table 1. Bibliographic reference lists of systematic reviews, which were dentified during screening, were searched to locate any studies that were not identified through the electronic literature database searches. To ensure unpublished RCTs missed through this process were not excluded, WHO International Clinical Trial Registry Platform (WHO-ICTRP), ClinicalTrials.gov, Cochrane Clinical Trial Register (CCTR), Australian New Zealand Clinical Trials Registry (ANZCTR), and EU Clinical Trials Register (EUCTR) were also searched using the same combination of keywords.

**Table 1:** PubMed and Web of Science search strategies

PubMed	
Search number	Search terms
1	Botulinum toxins[MeSH] OR Botulinum toxin*[tw] OR Botulin[tw] OR Clostridium botulinum toxin* [tw] OR Botulinum toxin type A[tw] OR “Clostridium botulinum A”[tw] OR “Clostridium botulinum type A”[tw]
2	Stroke[MeSH] OR Stroke[tw] OR strokes[tw] OR Apoplexy[tw] OR CVA[tw] OR CVAs[tw] OR “Cerebrovascular Accident”[tw] OR “Cerebrovascular Accidents”[tw] OR “Cerebrovascular Apoplexy”[tw] OR “Cerebrovascular Stroke”[tw] OR “Cerebrovascular Strokes”[tw] OR “Brain Vascular Accident”[tw] OR “Brain Vascular Accidents”[tw] OR “Cerebral Stroke”[tw] OR “Cerebral Strokes”[tw] OR “Acute Stroke”[tw] OR “Acute Strokes”[tw] OR “Acute Cerebrovascular Accident”[tw] OR “Acute Cerebrovascular Accidents”[tw]
3	Muscle Spasticity[MeSH] OR Spasticity[tw] OR Spastic[tw] OR Hypertonia[tw] OR “muscle overactivity”[tw]
4	#1 AND #2 AND #3
5	Filters - Species: Humans; Languages: English; Search Date: 16/9/2015
Web of Science	Search terms
1	Botulinum toxin or Botulin or Clostridium botulinum toxin or Botulinum toxin type A or Clostridium botulinum A or Clostridium botulinum type A
2	Stroke or Apoplexy or CVA or Cerebrovascular Accident or Cerebrovascular Apoplexy or Cerebrovascular Stroke or Brain Vascular Accident or Cerebral Stroke or Acute Stroke or Acute Cerebrovascular Accident
3	Spasticity or Spastic or Hypertonia or Muscle overactivity
4	#1 AND #2 AND #3
5	Filters - Species: Humans; Languages: English; Search Date: 16/9/2015

### Inclusion and exclusion criteria

The authors screened independently the title and abstract of studies, identified through the literature for potential inclusion. For this study, BoNTA (Botox® or Dysport® or Xeomin®) was defined to include any clinical use, of any dosage or duration, for the treatment of adult post-stroke lower limb spasticity (PSLLS). The review includes RCTs examining the use of BoNTA versus placebo use in adult PSLLS and included spasticity of any lower limb muscle of any severity or duration. The functional outcomes included were gait velocity, walking distance, and QOL measures.

The review excluded studies without a placebo-control group, observational studies using other types of BoNTA, or studies not reporting any of the outcomes mentioned above. Studies involving spasticity of non-stroke etiology, immobile patients, and patients with fixed contracture or pregnancy were also excluded.

### Data extraction

Two reviewers (ADG and WHC) independently extracted data from the included studies. Data included sample size, study design, intervention methodology, participant randomization, timing of intervention and follow up, and outcomes—including gait speed, walking distance, and/or QOL. Other functional outcomes of significance were also extracted.

### Assessment of methodological quality

Methodological quality of the studies was not addressed until the final selection of studies had been made for this systematic review. Methodological quality of individual studies was critically appraised using Joanna Briggs Institute’s instrument and included in our study analysis. Those with RCT scores ≥ 8 were included in the study (Table 2). This instrument mandates that the quantitative papers are selected for retrieval by two independent reviewers for methodological validity prior to inclusion in the review using standardized critical appraisal from the institute’s review instrument [19].

**Table 2:** Quality assessment of the included studies using Joanna Briggs Institute’s appraisal instrument

Study	Sample randomization^a^	Inclusion criteria^b^	Confounding factors/bias^c^	Outcome criteria^d^	Comparison group description^e^	Sampling timing^f^	Participants withdrawal^g^	Outcome measurement^h^	Data synthesis^i^	Inclusion^j^	Total score
Kaji et al. [22]	Yes	Yes	Unclear	Yes	Yes	Yes	No	Yes	Yes	Yes	8
Pittock et al. [23]	Yes	Yes	Yes	Yes	Yes	Yes	Yes	Yes	Yes	Yes	10
Tao et al. [20]	Yes	Yes	Yes	Yes	Yes	Yes	Unclear	Yes	Yes	Yes	9
Burbaud et al. [24]	Yes	Yes	Unclear	Yes	Yes	Yes	No	Yes	Yes	Yes	8
Johnson et al. [21]	Yes	Yes	Yes	Yes	Yes	Yes	No	Yes	Yes	Yes	9
Yes = 1; no/unclear = 0											

^a^Was the study based on a random or pseudo-random sample?

^b^Were the criteria for inclusion in the sample clearly defined?

^c^Were confounding factors identified and strategies to deal with them stated?

^d^Were outcomes assessed using objective criteria?

^e^If comparisons are being made, were there sufficient descriptions of the groups?

^f^Was follow-up carried out over a sufficient time period?

^g^Were the outcomes of people who withdrew described and included in the analysis?

^h^Were outcomes measured in a reliable way?

^i^Was appropriate statistical analysis used?

^j^Is this study to be included in the systematic review?

## Results

### Description of the included studies

No studies included in this review were excluded based on their quality scoring. Table 2 shows that accepted studies achieved a quality score of at least 8. Below this score, studies were excluded because they were not RCTs or did not include a placebo control.

**Fig. 1:**
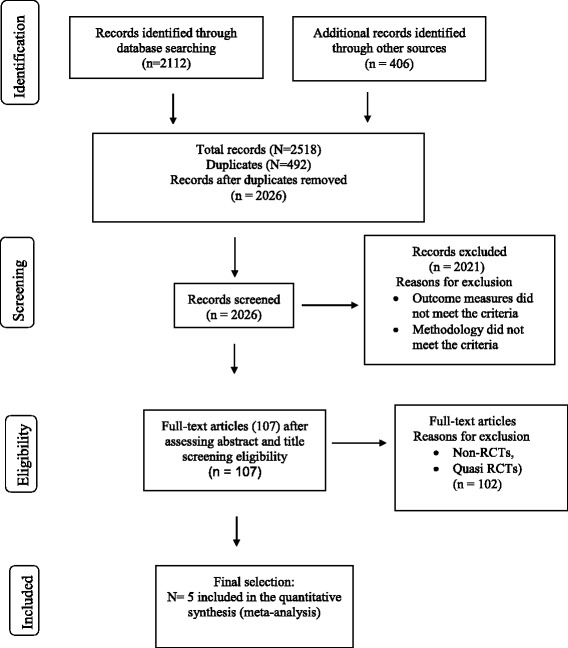
PRISMA flow diagram

Figure 1 illustrates the PRISMA diagram with all the RCTs and the flow chart for the reviewing process. We identified 2112 records through database searches and 406 records from other sources. After removing the redundant studies, we ended with 2026 records. On screening for title and abstracts, we found 107 full-text records. Amongst them, the number of non-RCT and quasi-RCTs was 102 and thus leaving five studies for further analysis. Table 2 summarizes the total scores of the five RCTs, which qualified for further analysis based on the quality assessment according to the predetermined criteria. Table 3 compares the main features including design, sample size, age group; exclusion criteria, power calculation, intervention, study results, and primary outcome measures that met the inclusion criteria.

**Table 3:** Comparison of five randomized controlled trials of botulinum toxin in post-stroke lower limb spasticity

Study characteristics	Tao W et al.	Kaji R et al.	Johnson C et al.	Pittock S et al.	Burbaud P et al.
Age group (years)	18–80	22–80	44–78	51–69	14–72
Primary outcome measure	Gait analysis Spasticity	MAS ankle score	Gait velocity	2 min Walking distance	Subjective rating of treatment efficacy
Sample size Study length Follow-up	*n* = 23 12 weeks 4 and 8 weeks	*n* = 120 12 weeks 4, 6, 8, and 12 weeks	*n* = 21 12 weeks 2, 4, 8, and 12 weeks	*n* = 234 12 weeks 4, 8, and 12 weeks	*n* = 23 4 months 0, 4, 12, and 16 weeks
Power calculation	None	Yes	None	Yes	None
Inclusion criteria	Post-stroke Ambulatory status not specified	Post-stroke Ambulatory status not specified	Post-stroke Patients were ambulatory	Post-stroke All patients could walk > 5 m	Post-stroke and trauma Non-homogenous Two patients could not ambulate
Intervention	EMG guided, 200 botox, gastrocnemius, soleus, tibialis	EMG guided, 300 botox, gastrocnemius, soleus, tibialis posterior	EMG guided, 1200 Dysport, gastrocnemius soleus, tibialis posterior	No EMG 500–1500 Dysport Gastrocnemius and soleus	EMG guided, 1000 Dysport, gastrocnemius, posterior, flexor digitorum longus
Statistical methods	Mean (SD), *t* tests, chi-square, Kruskal-Wallis	Mean (SD), *t* tests, area under curve, Wilcoxon	Mean (SD), ANCOVA, graphic plots	Mean (SD), one-way ANCOVA, Dunets test, Rank test	Mann-Whitney
Study results	Gait speed ⇧ Sig MAS ⇩Sig FMA ⇧ Sig MBI ⇧ Sig	Gait speed ⇨ N.S MAS ⇩ Sig CGI ⇨ N.S PRS ⇨ N.S	Gait speed ⇧ sig MAS ⇩ Sig, RMA ⇩ Sig, SF-36 ⇨ N.S	2-min walk ⇨ N.S Step length ⇨N.S Aids ⇨ N.S. Subjective ⇨ N.S MAS ⇩ Sig, RMA ⇨ N.S	MAS ⇩ Sig, FMA ⇩ sig, Spasticity ⇨ N.S
Secondary outcome measures	MAS, FMA, MBI, sEMG	Physician Rating Scale of gait, gait velocity, CGI, safety	MAS, PCI, RMA, SF-36	MAS, step length, RMA, active and passive range of ankle movement	MAS, FMA, gait velocity
Concurrent therapy	Standardized	Not standardized	Standardized	38% received therapy	Not standardized

*MAS* Modified Ashworth Scale, *FMA* Fugl-Meyer Assessment, *MBI* Modified Barthel Index, *sEMG* surface electromyograph, *CGI* Clinical Global Impression, *RMA* Rivermead Motor Assessment, *EMG* electromyograph, *Sig* significant, *N.S* not significant

### Quality of the included studies

Table 3 shows that each of the studies had a different primary outcome measure [20–24]. Variation between studies also extended to age group, inclusion criteria, and intervention protocol. Despite this, it can be seen from Table 3 that there were many promising outcomes. Tao et al. [20] showed gait analysis (step length, cadence, and speed) improved in the treatment group, and Johnson et al. [21] demonstrated treatment group changes in the effort of walking (speed walkway) and improvement in mobility. There were also improvements in spasticity (MAS) in all studies, though in the Johnson study, this may not be related to the intervention. Johnson et al. [21] also reported a non-significant improvement in the self-reported quality of life (SF-36). Despite the overall significant outcomes in various functional measures, there was little corroboration of improvement in functional outcomes across studies, and it was concluded that, given the range of variations in study measures between studies, a meta-analysis of the data should not be conducted. The decision is supported by the different inclusion criteria (e.g., age, ambulatory status, homogeneity) used in each study, which raises the question of sample comparability. Furthermore, although spasticity is clearly and successfully measured as an outcome in all studies, the methods used to assess walking ability varied between studies, and no more than two studies used the same methods with successful outcomes. This would not support a substantial meta-analysis, and in addition, further design issues were apparent. The potential for meta-analysis was also invariably compromised in studies assessing the same outcome, i.e., gait speed and MAS, because of small sample size and large confidence intervals around the estimate. In addition, where estimates for outcomes were positive, three studies did not report power calculations [20, 21].

As shown in Table 3, the absence of a power calculation in three of the studies is a serious design shortcoming. Kaji et al. [22] and Pittock et al. [23] were the only investigators to provide a power calculation. The largest study sample, which did include a power calculation, exceeded the smallest sample size by a factor of 10 [23]; this seriously questions the sample size calculations used in the smaller studies and therefore the reliability of estimates. For the remaining studies, it should be reinforced that a small sample size in studies reduces the chance of detecting a true effect but, in addition, low power (which is a characteristic of the smaller studies) also increases the chance of both type 1 and type 2 errors. Given the age ranges of the smaller study samples included in the present study, assumptions of study representativeness are seriously questioned, and consequently, if the distribution of the study sample is skewed, the *t* test (used in the smaller studies) is not appropriate. Reliability is also questioned in the Kaji et el. study [22], which was of short duration of only 12 weeks following long post-stroke period of 6 years. In addition, none of the studies used other important indices to assess functional outcomes such as balance (Berg Balance Score); Timed Up and Go (TUG), a test for mobility; or the Goal Attainment Scale (GAS), a patient perceived assessment of improvement in function.

Underpinning assessment of the quality of all these studies is the issue of repeated measures and assessment of temporal trends. The assessment of temporal trends was poorly handled. For the most part, change over time was assessed by comparing separately each time point to baseline (e.g., Pittock et al. [23]; week 4 to baseline, week 8 to baseline, and week 12 to baseline), and this approach suffers two limitations: first, time trends are not well characterized as this would require all time points to be assessed simultaneously; second, the approach fails to acknowledge that the outcome at one time in point may be influenced by earlier assessments. It is a known feature of repeated measures data that outcomes within patients are correlated, and the failure to account for this in the analysis has serious consequences. Variance estimates are likely to be biased (variance is underestimated) which increases the risk of reporting a type 1 error. Two studies applied alternative methods to characterize trends over time. Johnson et al. [21] used simple linear regression, although it is not clear how this was applied. Since these authors argued that mean differences across time points could not be directly compared, it would seem reasonable to assume that they entered each time point as separate covariates. Their solution to resolving these issues with the comparisons was to run an ANCOVA hence producing baseline adjusted effects for the final assessments. The use of simple linear regression and/or ANCOVA is inappropriate in this instance; observations are treated as independent (i.e., uncorrelated) observations resulting, again, in variance being underestimated with the consequential increased risk of reporting a type 1 error. Kaji et al. [22] attempted to characterize group differences in temporal trends for the primary outcome by analyzing area under the curve (AUC). It is not clear why this approach was taken as AUC is generally used for other purposes, for example, in pharmacology, to plot concentrations of drugs in blood plasma over time [25]. It is difficult to relate this to the purpose of the trial, which is to assess the efficacy and safety of the treatment, which, we conclude, is best assessed using other methods.

## Discussion

This review shows the evidence for functional improvement such as walking and QOL using BoNTA for PSLLS to be inconclusive, given the variations and shortcomings in study designs and methodologies. Thus, we argue meta-analysis should not be undertaken to assess the efficacy of BoNTA on functional outcomes. We would, however, conclude that the studies do provide some promising indications of improvement in functional outcomes that requires re-investigation in a well-planned RCT with substantial design and analytic changes. About design, appropriate sample calculations must be based on a repeated measures design. The optimum approach to analyze repeated measures data in future RCTs would be to apply repeated measures ANOVA or a linear mixed-effects model, unlike simple linear regression and ANCOVA as applied by Johnson et al. [20]. With these methodological changes, direct comparisons between groups and between time points can be made. They also account for patient correlations with appropriate adjustments to variance estimates hence reducing the risk of reporting a type 1 error. As already mentioned, power calculations were not reported in three studies, and given the small sample assessed, this is likely to be a significant limitation. Kaji et al. [22] did report power calculations and obtained a sample larger than the one required. However, the effect size reported was lower than that assumed for the calculations (3.428 vs. 5.0) with the consequent loss of statistical power. This slipped from 90 to 80% even with the addition of approximately 11 subjects per group. While 80% power is the standard for most studies, more power is often used in clinical trials and should be used in future RCTs. Pittock et al. [23] provided power calculations and recruited a sample that was larger than indicated by the calculations. However, the obtained effect size was not reported for the parameter that was used as the basis for the power calculation, and it is difficult to establish whether the requisite power was obtained although this was likely. Sample sizes need also to take account of stratification in the study analyses. Furthermore, BT injection is a focal injection, and the effect size from such injection is likely to be small, and hence, the optimal sample size needs to be calculated using minimum clinically important difference (MCID) in the primary outcome with adequate power.

Future RCTs should include homogenous post-stroke patients with similar baseline characteristics, intact cognition, and with some walking ability. It is unlikely that patients with significant PSLLS who are unable to move will achieve active functions such as walking post-BoNTA injection. Other studies need to be designed to consider improvements for these more severe post-stroke patients. Significant receptive aphasia impedes participation in rehabilitation, and these patients should be excluded. Best outcomes may be achieved if BoNTA is administered as early as two-week post-stroke [26].

Future studies should also consider gait velocity or 6-min walk test as primary outcomes as per International Classification of Functions (ICF) [27]. Only two studies in our review included this clearly. It is also necessary to document activity limitation and participation restriction according to the International Classification of Functioning, Disability and Health [27]. Oher functional outcomes should be considered for inclusion including balance (by Berg Balance Score), Time Up and Go (TUG), and the Goal Attainment Scale (GAS). Minimization of PSLLS may facilitate recovery of balance [28]. Studies should also consider economic analyses for cost-benefit purposes such as quality-adjusted life years (QALYs) from the quality of life measures. QALYs are measures of disease burden which includes both quality and quantity of life lived and a value of health outcome.

Accuracy of the intervention using BoNTA is also dependent on the use of injection guidance such as electromyograph (EMG) or ultrasound (US). In this review, only three studies did so. The involved muscles should be identified through functional activities such as walking where possible and confirmed by EMG or US prior to proceeding with injection for optimum result. It is important to understand that the equinus component of the equinovarus deformity is caused by the spastic gastrocnemius/soleus with contributions from the tibialis posterior and other long toe flexors, whereas a varus deformity is mostly caused by the tibialis posterior. It is important to identify and inject the specific muscles causing the deformity. Pittock et al. [23] failed to do this. BoNTA works in conjunction with other conventional therapies like physiotherapy and splinting. Once BoNTA has reduced the spasticity, patients need gait training to learn new motor control facilitating the speed of walking and walking distance. Evidence suggests that intensive repetitive practice with incremental difficulty within the tolerance limit can enhance walking ability [29]. A standardized physical therapy should be designed to address intensity, frequency, and duration in future RCTs.

Three other systematic reviews deserve mentioning before concluding. The first of these by Foley et al. [30] reviewed the effects of BoNTA on gait velocity on five RCTs and three uncontrolled studies using small samples and found a small improvement in gait velocity. Two of these studies were included in our review. The second by Baker et al. [31] assessed BoNTA effects on gait speed and quality of life and found no significant effect using two of the studies included in this review. More importantly, this review was not stroke specific. Most recently, Wu et al. [32] used seven studies to assess lower limb spasticity only. The present review included four of the studies included by Wu et al. This review included studies on lower limb spasticity of heterogeneous origin, and some studies were not placebo-controlled RCTs [32]***.***

### Limitations

The search was over 1 year old during preparation and submission. We conducted an additional verifying search on CINHAL, EMBASE, and MEDLINE (via PubMed) from 16 September 2015 to 6 June 2017 using the same clauses as the previous search, and no relevant trial was identified.

The review was limited by the inability to conduct meta-analysis of the data. We employed very strict criteria in the selection of the included RCTs and excluded many uncontrolled studies. Significant heterogeneity of outcomes limited the ability to draw firm conclusions, and we were unable to test publication bias.

## Conclusions

In conclusion, this systematic review has demonstrated the need for further research into the use of BoNTA for PSLLS. There is a significant lack of well-designed RCTs assessing functional improvement in PSLLS post-BoNTA injection. It is important therefore to invest in a well-designed BoNTA study to investigate more thoroughly the effects on active functioning such as walking and functional ability in PSLLS. The compound has already demonstrated the ability to reduce spasticity, but to argue, BoNTA is a standard treatment for PSLLS, its effects on walking and functional ability must be established. It is highly recommended that study initiatives should be directed toward achieving functional outcomes and participation goals [33]. We propose an adequately powered placebo-controlled study of BoNTA on a homogenous group of patients with PSLLS who have some walking ability. The study should have the sample size calculated from the MCID of the primary functional outcome of gait velocity or other important functional outcomes including QOL, which may require larger sample size than used in studies to date. Appropriate muscle selection and targeted injection, standardized adjuvant therapy, and proper statistical methods are essential in finding the benefits on the lower-limb functions. Finally, we return to the purpose of using BoNTA for lower limb functionality. If the aim of such studies is only to reduce spasticity, this is already proven, the real challenge is improving function and quality of life. This review has informed us to design our own RCT (currently underway) in our hospital looking at the improvement of lower limb functioning such as walking and quality of life in post-stroke lower limb spasticity using botulinum toxin [34].

## Abbreviations

AUC: Area under curve
BoNTA: Botulinum toxin A
CGI: Clinical Global Impression
EMG: Electromyograph
FMA: Fugl-Meyer Assessment
MAS: Modified Ashworth Scale
MBI: Modified Barthel Index MCID Minimally clinically important difference
N.S: Not significant
PBS: Pharmaceutical Benefit Scheme
PSLLS: Post-stroke lower limb spasticity
QOL: Quality of life
QALY: Quality-adjusted life years
RCT: Randomized control trial
RMA: Rivermead Motor Assessment
sEMG: Surface electromyograph
Sig: Significant
TUG: Timed Up and Go
US: Ultrasound
